# COMMON BARRIERS AND FACILITATORS ACROSS DIVERSE DOMAINS OF AGE-RELATED PLANNING

**DOI:** 10.1093/geroni/igz038.1588

**Published:** 2019-11-08

**Authors:** Emily K Chen, Emily K Chen, Daniel Siconolfi

**Affiliations:** 1 RAND Corporation, Arlington VA, Virginia, United States; 2 RAND Corporation, Arlington, Virginia, United States; 3 RAND Corporation, Pittsburgh, Pennsylvania, United States

## Abstract

Advance care planning (ACP) – plans that express wishes for healthcare for when a person is unable to communicate – is often studied independently of other age-related planning activities. This study explored a broad set of age-related planning activities, such as retirement, finances, aging in place, and healthcare, including ACP. We used directed content analysis to identify barriers and facilitators to age-related planning from semi-structured interviews with 38 respondents (ages 55-74). Surprisingly, a common set of structural, interpersonal, and individual barriers and facilitators emerged across domains. Barriers included competing demands, resistance from family members, and aversion to planning in general. Facilitators included exposure to planning behaviors through professional and social networks, having witnessed negative outcomes from others’ failure to plan, and a belief that planning would spare others future distress. These results reinforce the idea that ACP exists within of a set of age-related planning behaviors that share common characteristics.

